# Harnessing CRISPR-Cas9 for Genome Editing in Streptococcus pneumoniae D39V

**DOI:** 10.1128/AEM.02762-20

**Published:** 2021-02-26

**Authors:** Dimitra Synefiaridou, Jan-Willem Veening

**Affiliations:** a Department of Fundamental Microbiology, Faculty of Biology and Medicine, University of Lausanne, Lausanne, Switzerland; University of Tartu

**Keywords:** CRISPR, Cas9, genome editing, *Streptococcus pneumoniae*, plasmids

## Abstract

CRISPR-Cas systems provide bacteria and archaea with adaptive immunity against viruses and plasmids by the detection and cleavage of invading foreign DNA. Modified versions of this system can be exploited as a biotechnological tool for precise genome editing at a targeted locus. Here, we developed a replicative plasmid that carries the CRISPR-Cas9 system for RNA-programmable genome editing by counterselection in the opportunistic human pathogen Streptococcus pneumoniae. Specifically, we demonstrate an approach for making targeted markerless gene knockouts and large genome deletions. After a precise double-stranded break (DSB) is introduced, the cells’ DNA repair mechanism of homology-directed repair (HDR) is exploited to select successful transformants. This is achieved through the transformation of a template DNA fragment that will recombine in the genome and eliminate recognition of the target of the Cas9 endonuclease. Next, the newly engineered strain can be easily cured from the plasmid, which is temperature sensitive for replication, by growing it at the nonpermissive temperature. This allows for consecutive rounds of genome editing. Using this system, we engineered a strain with three major virulence factors deleted. The approaches developed here could potentially be adapted for use with other Gram-positive bacteria.

**IMPORTANCE** Streptococcus pneumoniae (the pneumococcus) is an important opportunistic human pathogen killing more than 1 million people each year. Having the availability of a system capable of easy genome editing would significantly facilitate drug discovery and efforts to identify new vaccine candidates. Here, we introduced an easy-to-use system to perform multiple rounds of genome editing in the pneumococcus by putting the CRISPR-Cas9 system on a temperature-sensitive replicative plasmid. The approaches used here will advance genome editing projects in this important human pathogen.

## Abstract (precis)

Streptococcus pneumoniae (the pneumococcus) is an important opportunistic human pathogen killing more than 1 million people each year. Having the availability of a system capable of easy genome editing would significantly facilitate drug discovery and efforts to identify new vaccine candidates.

## INTRODUCTION

Streptococcus pneumoniae (the pneumococcus) is a Gram-positive human commensal that colonizes asymptomatically the mucosal surfaces of the upper respiratory tract (URT) (1). However, in susceptible groups such as children, the elderly, and the immunocompromised, it can occasionally cause disease, ranging from a mild URT infection, acute otitis media, and sinusitis to severe and potentially life-threatening conditions such as pneumonia, bacteremia, and meningitis (2). It is responsible for more than 1 million deaths annually (3), and in 2017, the World Health Organization (WHO) classified S. pneumoniae as one of 12 priority pathogens for which new antibiotics are urgently needed.

Historically, S. pneumoniae research played a central role in advancing molecular biology. While trying to develop a vaccine against the pneumococcus, Griffith discovered natural transformation (4). This was followed by research of Avery, MacLeod, and McCarty to establish that DNA is the genetic material (5). Over the last decade, the pneumococcus has become a valuable model to study the cell biology of ovoid-shaped bacteria, and several cell biological tools such as integration vectors, fluorescent reporters, inducible promoters, and CRISPR interference have been established for this organism (6–8). In addition, many selection and counterselection methods are available, making it relatively easy to generate gene deletions, gene complementation mutants, or point mutations in the pneumococcal genome (9–12). However, all current gene deletion methods established for S. pneumoniae are poorly scalable and often require a specific genetic background to function (e.g., the *rpsL^+^* background in the Janus system) (9).

In the case of gene replacement by selection markers, while powerful, this also has drawbacks, preventing further modifications of the genome when there are no further selectable markers available for additional strain development. Also, many important categories of gene mutation, such as missense substitutions and in-frame deletions, usually present no selectable phenotype (9). To circumvent these issues, we here established CRISPR-Cas9 genome editing for use as counterselection in the pneumococcus.

Clustered regularly interspaced short palindromic repeats (CRISPR) and CRISPR-associated (Cas) proteins are present in many bacteria and most archaea (13). Naturally, the system provides resistance against foreign genetic elements (e.g., phages or plasmids) via small noncoding RNAs that are derived from CRISPR loci (14). In class 2 type II CRISPR systems, the mature CRISPR RNA (crRNA) that is base paired to a *trans*-activating crRNA (tracrRNA) forms a two-RNA structure that directs the CRISPR-associated proteins (e.g., Cas9 from Streptococcus pyogenes) to introduce a double-stranded break (DSB) into the target DNA locus. Site-specific cleavage occurs at locations determined by both base-pairing complementarity between the crRNA and the target protospacer DNA and a short protospacer adjacent motif (PAM) (15). It has been demonstrated that the endonuclease can be programmed by engineering the mature dual-tracrRNA: crRNA as a single RNA chimera (single guide RNA [sgRNA]) to cleave specific DNA sites. Thereby, modified versions of the system can be exploited as a biotechnological tool for precise, RNA-programmable genome targeting and editing (15).

After the DSB has been introduced by Cas9, the cell can utilize two major pathways in order to repair the break and survive: homologous recombination (HR) or nonhomologous end joining (NHEJ). In HR, a second intact copy of the broken chromosome segment, homologous to the DSB site, serves as a template for DNA synthesis across the break. In this mechanism, the crucial process of locating and recombining the homologous sequence is performed by RecA (16). NHEJ does not rely on a homologous DNA template, as the two DNA ends are rejoined directly together. Most bacteria, including S. pneumoniae, cannot perform NHEJ but are capable to perform HR (17). DSB repair can be used as a way to generate mutants or desired changes to the genome by providing an HR template and forms the basis of CRISPR-Cas engineering (18). Indeed, early work, using integrative vectors and tracrRNAs, showed that Cas9 can be used to make markerless gene deletions in S. pneumoniae (19).

In this study, we set out to establish a more concise CRISPR-Cas engineering framework for S. pneumoniae harnessing Cas9 from S. pyogenes. Specifically, we constructed a replicative plasmid containing a temperature-sensitive origin of replication (facilitating curing of the plasmid) carrying a genetic system for making targeted, markerless gene knockouts and large genome deletions, which works with high efficiency in S. pneumoniae. The plasmid system developed here could potentially be transportable to other Gram-positive bacteria, as the used origin of replication was shown to be functional in Lactococcus lactis and Bacillus subtilis (20). While similar approaches have recently been undertaken to perform genome engineering in certain Gram-positive organisms such as Enterococcus faecium (21), *Clostridium* (22), Lactobacillus reuteri (23), Lactobacillus plantarum (24), and Lactococcus lactis (25), a concise CRISPR sgRNA:Cas9 gene editing system was not yet available for S. pneumoniae, and the vector described here has the advantage of being readily curable due to its temperature-sensitive origin of replication.

## RESULTS

### An S. pneumoniae replicative plasmid that carries the CRISPR-Cas9 system.

**FIG 1:**
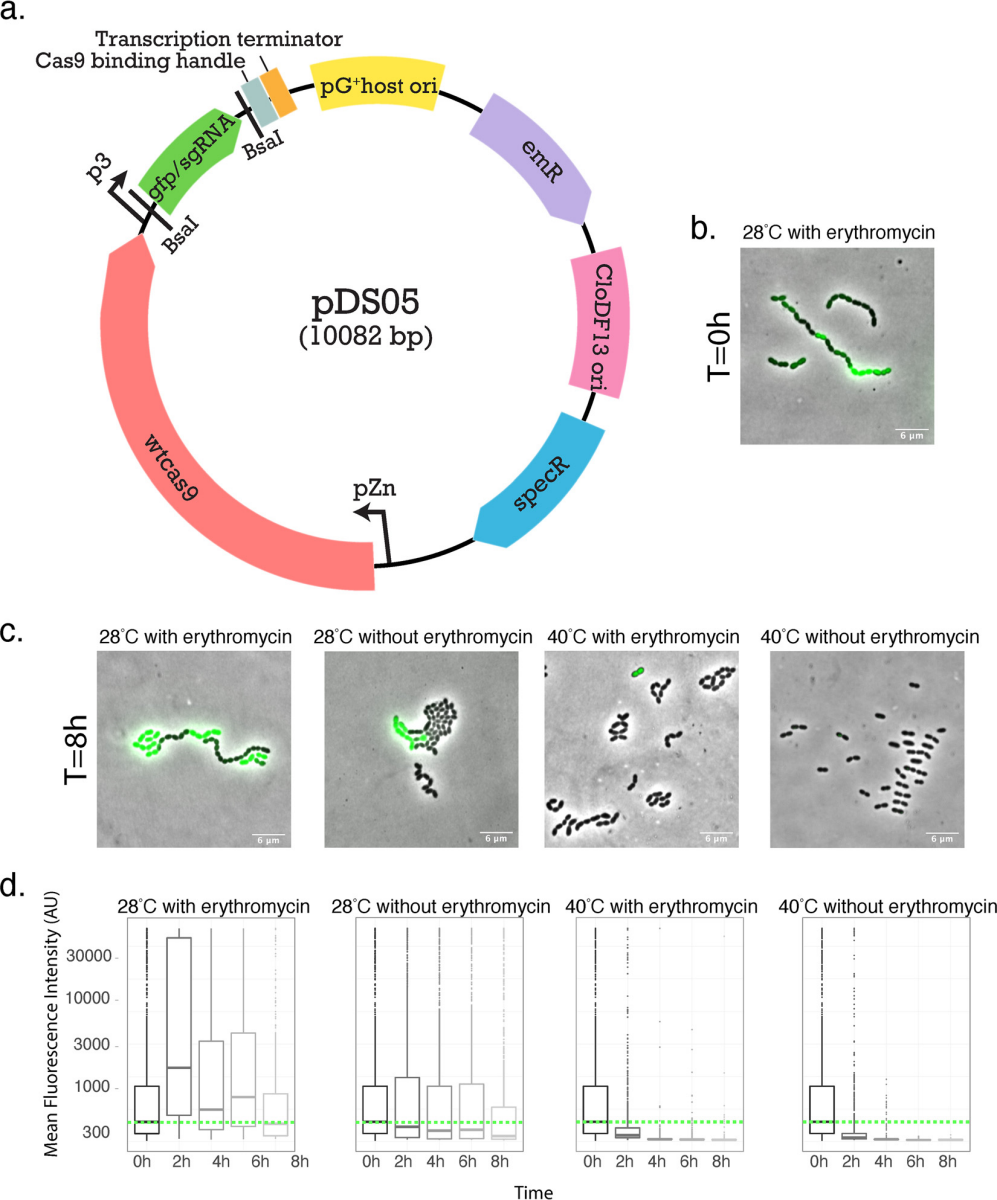
pDS05 is a temperature-sensitive plasmid that can be used for CRISPR-Cas9 genome editing in S. pneumoniae. (a) Schematic representation of plasmid pDS05. (b) Microscopy analysis of strain VL3655 [D39V(pDS05]. Overlay of GFP signals with phase-contrast image shows GFP expression. Note that the levels of fluorescence have been adjusted because of the large cell-to-cell variability in fluorescence (some cells appear dark but actually produce above-background levels of fluorescence). Preculture grown at 28°C with erythromycin (*T* = 0 h), (c) Images are shown of cells grown for 8 h as exponentially growing cells (balanced growth) under four different conditions: 28°C with erythromycin, 28°C without erythromycin, 40°C with erythromycin, and 40°C without erythromycin. (d) Quantification of mean fluorescence intensity of GFP of cells grown under four different conditions: 28°C with erythromycin, 28°C without erythromycin, 40°C with erythromycin, and 40°C without erythromycin at time points of 0, 2, 4, 6, and 8 h after dilution from the 28°C with erythromycin condition. Fluorescence microscopy of approximately 1,000 cells per condition per time point were quantified and analyzed using MicrobeJ and BactMAP and plotted as box plots (box size and line represent the average intensity per cell) (see Materials and Methods). The green dotted horizontal lines indicate the mean fluorescence of cells from the preculture harboring pDS05.

First, a replicative plasmid was designed and constructed (Fig. 1a) by combining PCR-amplified genomic and plasmid parts. The main idea behind the choice for individual vector components relied on creating a platform with the CRISPR-Cas9 system in S. pneumoniae while at the same time allowing for plasmid propagation in both Gram-positive and Gram-negative hosts. The modular vector consists of six individual components and two origins of replication: the high-copy-number p*G^+^host* replicon, which is a replication thermosensitive derivative of pWV01 (26) that, in L. lactis (and other Gram-positive bacteria), replicates at 28°C but is lost at temperatures >37°C, and the low-copy-number *cloDF13* (CDF) replicon for propagation in Escherichia coli. By combining these two origins of replication, it ensures low copy numbers at 37°C in E. coli, thereby preventing toxicity of the CRISPR-Cas9 system while cloning. Additionally, it has the gene which encodes wild-type Cas9 (wtCas9) under the control of the zinc-inducible promoter *Pzn* (27) (plasmid pDS05) and genes conferring spectinomycin (E. coli) and erythromycin (S. pneumoniae) resistance. Finally, it has the strong synthetic constitutive *P3* promoter, which was optimized before specifically for S. pneumoniae (28). Additionally, since analysis of the nucleotide distribution across all transcription start sites (TSSs) showed a strong preference for adenine as the initiating nucleotide in S. pneumoniae (29), we have added an adenine as +1 for the sgRNA. The *P3* promoter is driving the sgRNA sequence in which the 20-base-pairing region of the sgRNA is replaced by the *gfp* gene flanked by two BsaI restriction sites. This allows for replacement of *gfp* by the spacer sequence of sgRNA with golden gate cloning. This way, successful cloning of the sgRNA allows for easy selection by absence of green fluorescent protein (GFP) fluorescence, giving us a versatile vector for different sgRNAs (see below).

### Successful plasmid propagation and plasmid curing in S. pneumoniae.

To test that the newly constructed plasmid was being replicated and genes were expressed in S. pneumoniae, we grew strain VL3655 (carrying plasmid pDS05) (Fig. 1a), which encodes GFP, in C+Y medium at 28°C in the presence of erythromycin. Fluorescence microscopy demonstrated that all cells produced GFP, although significant cell-to-cell variability was observed (Fig. 1b). GFP intensity levels were determined in exponentially growing cells. Additionally, cells pregrown at 28°C in the presence of erythromycin (time [*T*] = 0) were split and grown under four different conditions: the permissive 28°C condition both with and without erythromycin in the growth medium and the nonpermissive 40°C with and without erythromycin. Note that growth was balanced by rediluting exponentially growing cells several times. Cells were collected every 2 h for 8 h; GFP intensity levels were determined using fluorescence microscopy, and images were analyzed using MicrobeJ and BactMAP (30, 31) (Fig. 1c and d). The results show that GFP levels and, by extension, plasmid copy numbers stayed stable at 28°C with erythromycin and slowly decreased in the absence of antibiotic pressure. Furthermore, GFP levels decreased significantly in cells grown at 40°C, confirming that this is a nonpermissive temperature for propagation of the plasmid. Absence of antibiotic pressure seems also to facilitate the decrease of the intensity levels of GFP, suggesting that the plasmid gets eliminated successfully under these conditions.

Since differences in plasmid copy number between cells can affect the levels of fluorescence, an additional time course experiment was conducted, with cells growing under the same conditions as mentioned above, and we determined the cells that are resistant to erythromycin compared to the total viable cells by counting the CFU (see Fig. S1a in the supplemental material). The results show that at the permissive temperature, most viable cells were erythromycin resistant, suggesting that they maintained the plasmid. Small discrepancies observed can be explained by the lack of separation of cells within the counted colony. At the nonpermissive temperature, none of the cells were resistant; therefore, they had lost the plasmid, supporting the previous microscopy findings. Finally, we also wanted to test whether Cas9 expression contributes to pDS05 plasmid instability. Therefore, we performed another fluorescence microscopy experiment (Fig. 1Sb), at the permissive temperature with and without antibiotic selection and with and without Cas9 induction by Zn^2+^. As shown in Fig. S1b, GFP levels and, by extension, plasmid copy numbers stayed stable under all tested conditions, suggesting that Cas9 induction in the absence of a targeting sgRNA does not cause plasmid loss.

### CRISPR-Cas9-mediated counterselection.

**FIG 2:**
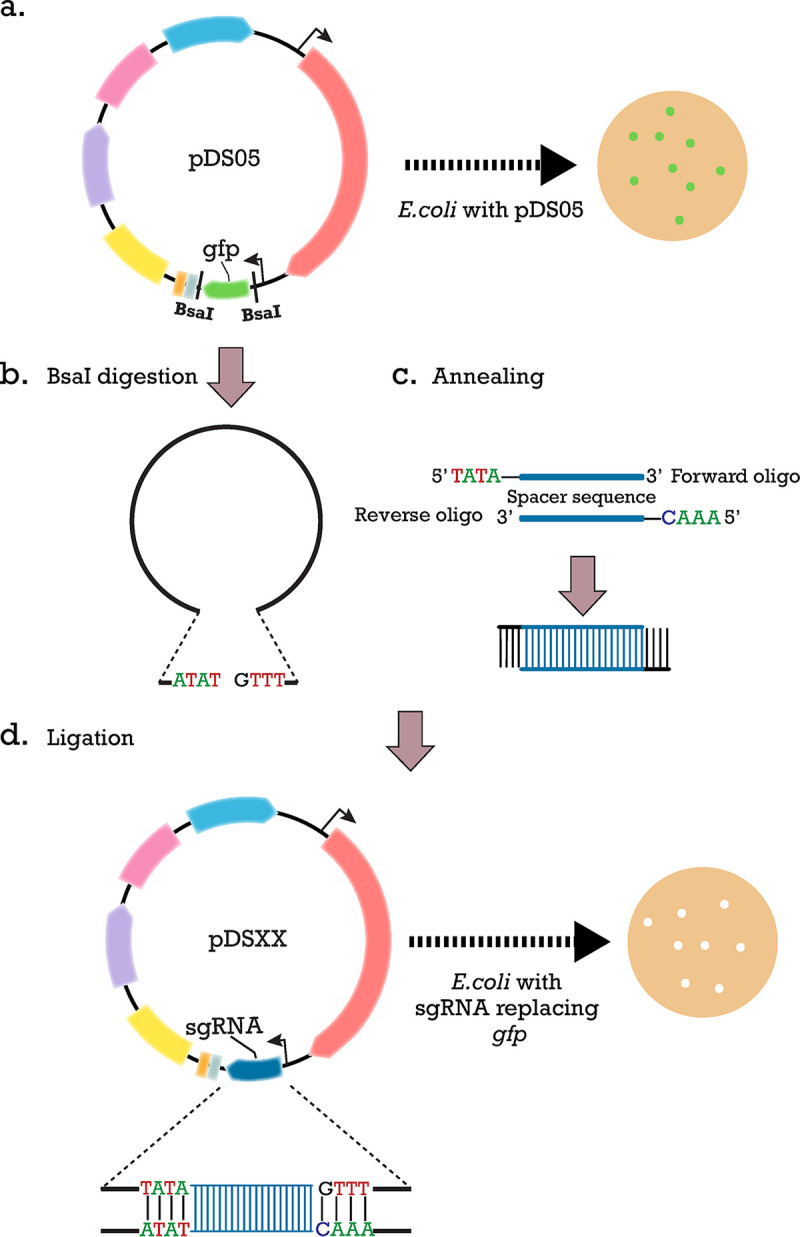
Workflow of sgRNA cloning. (a) pDS05 was designed to facilitate easy replacement of *gfp* by the spacer sequence of the desired sgRNA with golden gate cloning, allowing also for detection of false-positive transformants. *gfp*, which encodes a green fluorescent protein, is in place of the spacer sequence of sgRNA and flanked by BsaI sites. E. coli with pDS05 produces green fluorescent colonies. (b) BsaI digestion of the vector exposes 4-nucleotide (nt) overhangs. (c) For each sgRNA, forward and reverse oligonucleotides were designed, as a reverse complement of each other, which after being annealed together, contained the 20-bp spacer sequence and 4-nt overhangs that can be specifically annealed with the digested vector. (d) Ligation of the digested vector with the sgRNA annealed product was transformed into E. coli, producing white colonies.

Once a deletion target has been selected, the plasmid with the specific sgRNA needs to be constructed. For the sgRNA design, several tools are available, but here, the freely available online software Benchling was used (Biology Software, 2019). Benchling uses the latest algorithms to instantly assess off-target effects and on-target efficiency for guide RNAs. Via this platform, we specified the guide parameters, which are a single guide of 20-bp length and an NGG PAM. From the list generated, sgRNAs without predicted mismatches and off targets were selected. As a second validation, the selected sgRNAs were evaluated using the R tool “CRISPRi-seq target validation” (32). The targeting of Cas9 to a locus of interest is achieved by cloning two annealed 24-bp DNA oligonucleotides (containing the 20-bp protospacer element) into the sgRNA backbone that matches the specified locus. First, *gfp* is removed from pDS05 by digesting with BsaI. Complementary oligonucleotides that carry the spacer sequence are annealed together. They are designed in a way that after annealing, they have overhangs complementary to those left after digestion of the backbone, as previously described (32) (Fig. 2). The desired plasmid is obtained after ligation and transformed into E. coli. False-positive transformants are easily identified, since they still carry the *gfp* and produce detectable fluorescent green colonies.

**FIG 3:**
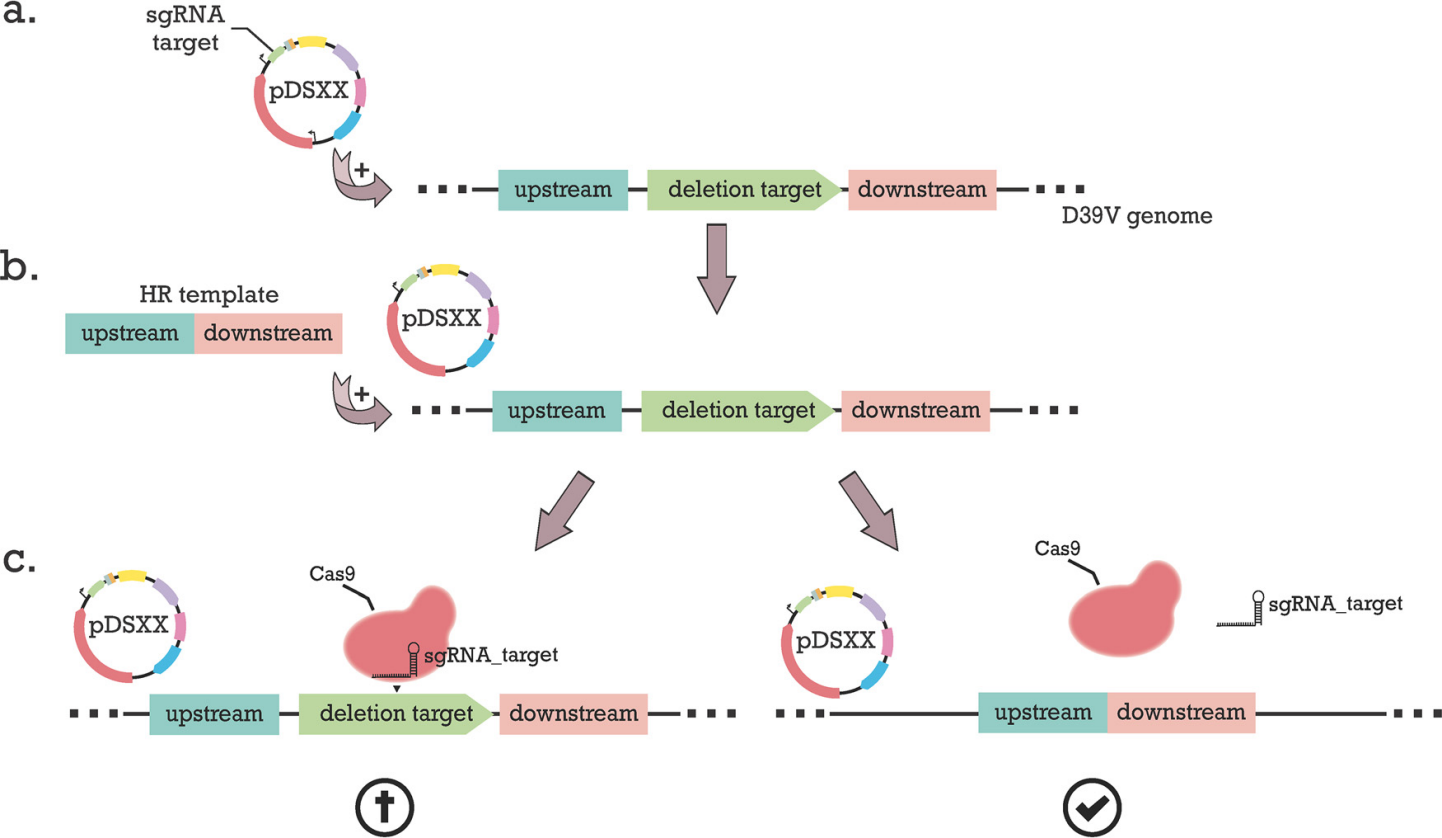
Workflow for markerless deletions. (a) Uptake of the plasmid by the strain with the sgRNA sequence for the desired deletion. (b) Transformation of a homology recombination (HR) template consisting of a fusion between the upstream and downstream regions of the deletion target. (c) Plating transformants with Zn^2+^ to induce expression of Cas9. Only the cells that have taken up and integrated the HR template eliminating the recognition target are able to survive.

After isolating the plasmid from E. coli, it needs to be used to transform S. pneumoniae. To achieve this, pneumococcal competence for transformation is induced by adding synthetic competence stimulating peptide (CSP). Note that competence-dependent transformation with a replicative plasmid is less efficient than transforming with linear homologous DNA (33), and so transformation efficiencies with pDS05 are typically low, with no evidence of differences between plasmids containing sham-sgRNA versus sgRNA targeting the chromosome when Cas9 is not induced (see Fig. S2). Next, an HR template is constructed that consists of the upstream and downstream regions of the deletion target. It has been shown that higher transformation efficiency is observed at higher donor DNA concentrations and with longer homology regions, with a plateau of approximately 1,000 bp of homology (34, 35). Therefore, each selected homology arm is designed to be at least 1,000 bp, ensuring efficient homologous recombination. Following again induction of competence by CSP, the pneumococcal cells harboring the pDS05 derivative are transformed with this template. Transformants are selected by plating with ZnCl_2_-MnSO_4_, the inducer of Cas9, offering CRISPR-Cas9-mediated counterselection. Only cells that have taken up and integrated the HR template, and thereby eliminated the recognition target of the sgRNA/Cas9 complex, would be able to survive, while untransformed cells will undergo DNA cleavage and die (Fig. 3).

### Deleting genes and large chromosomal regions from the S. pneumoniae genome.

**FIG 4:**
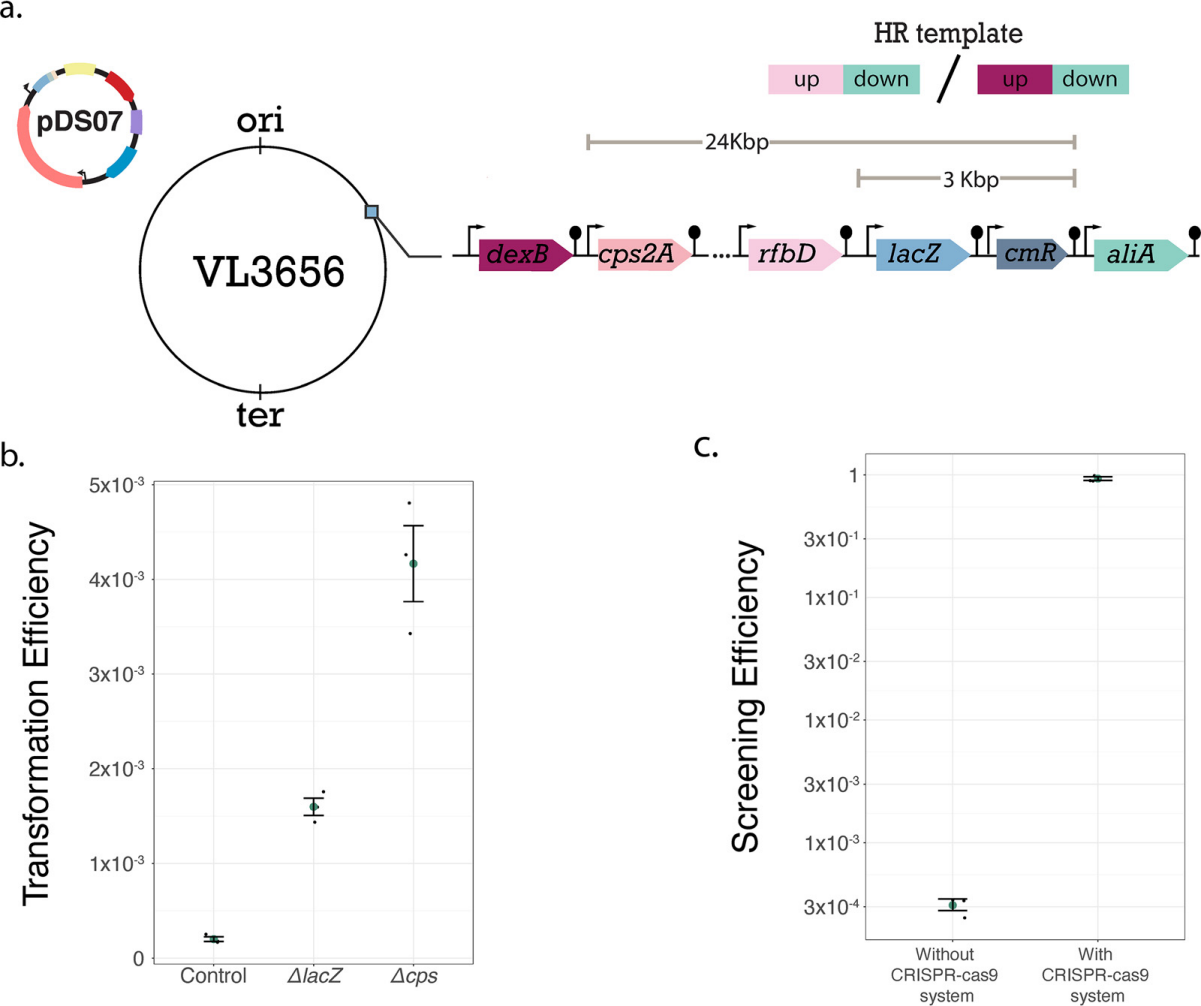
Genome editing in S. pneumoniae using CRISPR-Cas9. (a) Schematic representation of strain VL3656. The *lacZ* gene has been inserted downstream of the capsule operon, and a version of the plasmid with an sgRNA targeting *lacZ* has been transformed into the strain. (b) Transformation efficiency of *lacZ* and capsule operon deletion. The transformation efficiency was calculated by dividing the total number of cells as counted on plates without Cas9 inducer (1 mM ZnCl_2_-MnSO_4_) by the number of colonies, which were also green, in the presence of inducer. Control is the transformation assay of strain VL3656 in the absence of HR template DNA. (c) Efficiency of successful transformants screened for integration of the *lacZ* deletion when using no selection and when using the CRISPR-Cas9 system. Data represent the averages from three independent experiments (± standard errors [SEs]).

To assess the efficiency of the system, we first constructed a strain (strain VL3656) (Fig. 4a) in which we placed the E. coli *lacZ* gene under a constitutive promoter behind the S. pneumoniae D39V *cps* locus (encoding the capsule). *lacZ* encodes a β-galactosidase that hydrolyzes X-Gal (5-bromo-4-chloro-3-indolyl-β-d-galactopyranoside) to produce a blue product, allowing for “blue/green” screening on plates. Colonies with blue color would still carry the *lacZ* gene, while colonies with the standard green (inside blood agar) color would indicate that the gene has been deleted from the chromosome.

Strain VL3656 also carries the pDS07 plasmid, which contains an sgRNA targeting *lacZ*. Next, we constructed an HR template that consisted of the 1,000-bp upstream and 1,000-bp downstream regions of *lacZ* (excluding *lacZ*), and we transformed VL3656 with this template (Fig. 4a). Transformants were selected by plating inside agar containing ZnCl_2_-MnSO_4_ to induce expression of Cas9.

After transformation of strain VL3656 with the HR template, transformation efficiencies were calculated (Fig. 4b). The CRISPR-Cas9-mediated counterselection, offered by the system, worked successfully. The selection efficiency was high, around 1.5 × 10^−3^, and almost all colonies in the transformation where the HR template was given and Cas9 was induced had their original green color, indicating the *lacZ* gene had been successfully deleted.

Eight transformants were tested for correct deletion of *lacZ* by colony PCR. The primers used were binding 1,000 bp upstream and downstream of *lacZ*, setting the correct PCR product of the successful deletion at 2,000 bp (5,000 bp if *lacZ* was not deleted) (see Fig. S3). All tested colonies had the expected product of 2,000 bp, demonstrating successful deletion of *lacZ*, resulting in strain VL3657 (Fig. S3). We note that, typically, selection of two or three transformants is sufficient, since editing efficiency is >99% (Fig. 4).

Additionally, we also used the system to delete an even larger chromosomal fragment. For this, we targeted the operon that encodes the capsule and the *lacZ* gene that had been inserted downstream of it, which is around 24-kbp long, allowing for blue/green screening. Once again, selection efficiency was very high, approximately 4 × 10^−3^, and almost all colonies had their original green color (Fig. 4b). Colony PCR verified correct deletion of the *cps-lacZ* chromosomal region (see below).

Using the same HR template to delete *lacZ*, we also performed transformation assays without the counterselection offered, by inducing our CRISPR-Cas9 system, and we performed again a blue/green color colony screening (Fig. 4c). More than 12,000 colonies needed to be screened to find three successful transformants with the original colony color among the blue colonies. In contrast, by using the system, almost with an absolute success rate, all the colonies on our plates were correct transformants, demonstrating how efficient our system is to easily select edited cells.

### Consecutive deletions of virulence factors of S. pneumoniae.

Once the capsule operon and *lacZ* were removed from the chromosome, it was confirmed by colony PCR. All tested colonies demonstrated the expected PCR product. One such colony was picked, resulting in strain VL3659. Next, we grew the new strain at the nonpermissive temperature (40°C), eliminating the plasmid, resulting in strain VL3660 (Δ*cps*).

**FIG 5:**
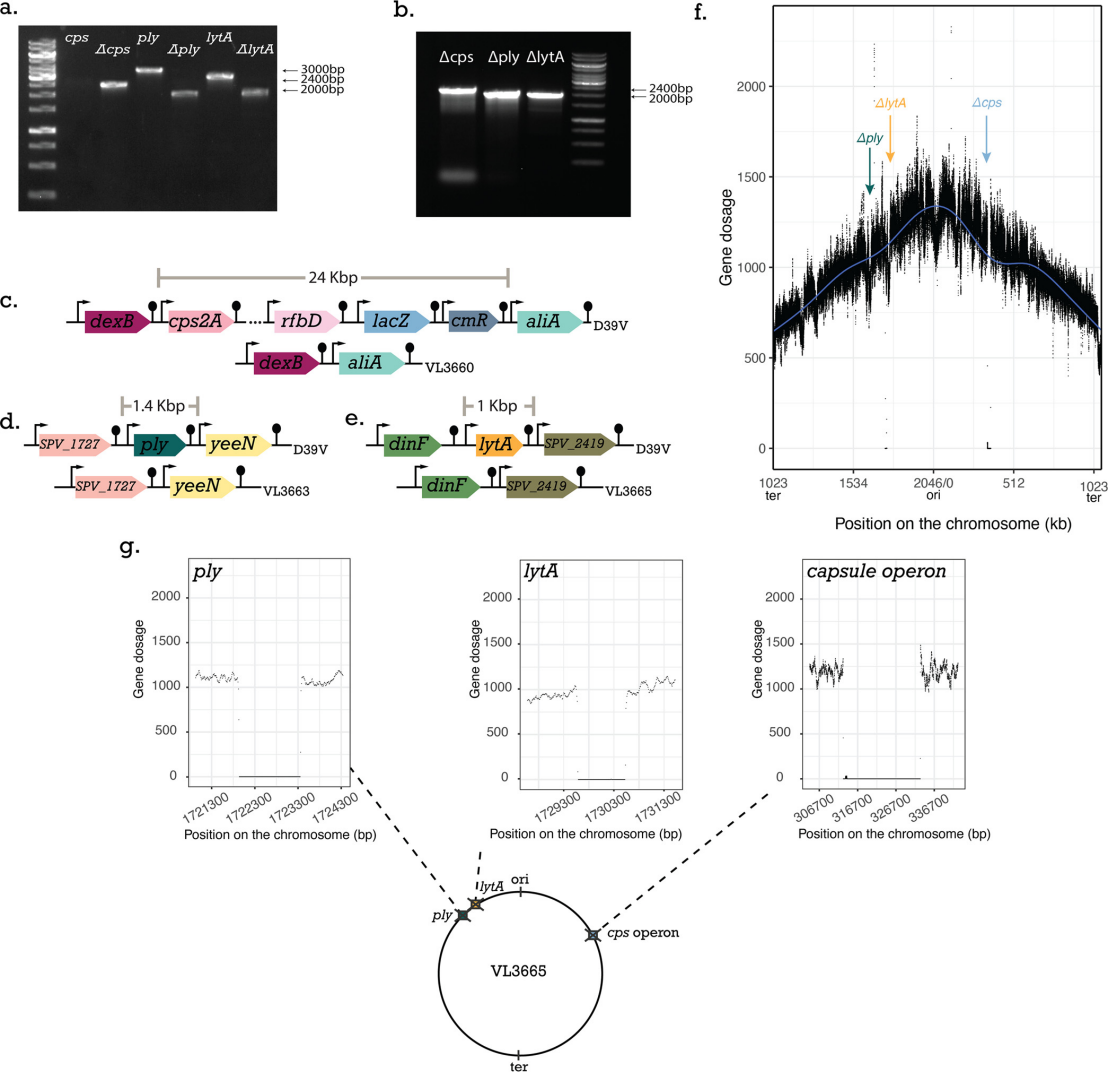
Genome analysis of the Δ*cps* Δ*ply* Δ*lytA* triple mutant generated using CRISPR-Cas9 editing. (a) Colony PCR analysis of expected sizes for deletion of three virulence genes. Wild type (WT) versus VL3665. (b) Colony PCR analysis of three virulence gene deletions in the final strain VL3665. (c) Schematic representation of the D39V capsule operon region before deletion and after deletion, resulting in strain VL360. (d) Schematic representation of the *ply* region. (e) Schematic representation of the *lytA* region. (f) Whole-genome marker frequency analysis of strain VL3665. The number of mapped reads (gene dosage) is plotted as a function of the position on the circular chromosome. (g) Schematic representation of strain VL3665 with three virulence genes deleted. A zoom in of 10 kb upstream and downstream of the deleted regions is shown.

To examine whether the system could be used in multiple rounds of genome editing, we attempted to delete the virulence factor pneumolysin. To delete the *ply* gene, we designed an sgRNA targeting pneumolysin and constructed plasmid pDS12, which we transformed into VL3660. Following the same procedure as used to delete the *cps* operon, we deleted *ply*. Again, to confirm the successful deletion, the same principle for the primer set was used. All the colonies from the transformation plate had the expected PCR product, demonstrating high selection efficiency using the CRISPR-Cas9 system. Finally, following the same strategy, we also deleted another important virulence factor, *lytA*, resulting in strain VL3665 [Δ*cps* Δ*ply ΔlytA*(pDS13)] (Fig. 5).

Cas9-dependent genome editing was specific without evidence for off-target cutting in constructing a triple deletion S. pneumoniae mutant.

After three consecutive deletions, using our plasmid with the CRISPR-Cas9 system, the final result was strain VL3665. It was previously shown that Cas9 tolerates mismatches between guide RNA and target DNA at different positions in a sequence-dependent manner, resulting in off-target DSBs (36). To examine the fidelity of our CRISPR-Cas9 system and whether there were detectable genome-wide off-target effects, we performed whole-genome sequencing (WGS). The analysis detected one single nucleotide polymorphism (SNP) in the genome, in the gene *psaA* (*SPV_1463*), encoding a manganese ABC transporter. The mutation results in a D137E amino acid change. Using Sanger sequencing, we confirmed that this SNP occurred only in the last strain of the consecutive deletions, and it was not present in the intermediate steps. This could have occurred as a result of needing to grow in the presence of ZnSO_4_-MnSO_4_ in the medium (37). However, comparing the growth of this strain with the ones that do not have the mutation, we do not observe any difference in fitness (Fig. S3). Furthermore, there is no evidence to indicate that this mutation is associated with an off-target effect, as the sequence surrounding the SNP is completely different from the used sgRNA present in plasmid pDS13. Therefore, it is likely that this SNP occurred randomly during growth, without affecting fitness. We do note that the WGS approach only assesses off targeting for the carefully selected three sgRNAs, and it remains to be seen how specific Cas9 is with suboptimal sgRNAs in S. pneumoniae.

Colony PCR on strain VL3665 confirmed that the three major virulence factors were deleted from the chromosome (Fig. 5a to e). Additionally, reads from whole-genome sequencing were competitively mapped onto the reference genome, our wild-type lab strain D39V (Fig. 5f). Direct comparisons between the genomes revealed the three chromosomal positions at which the deletions have taken place, since at these positions, the read coverage drops. Therefore, we confirmed that we had successfully performed markerless deletions of these three genes (Fig. 5g).

## DISCUSSION

Genetic manipulation of microorganisms has been pivotal for the development of biotechnological tools and the study of microorganisms themselves. CRISPR-Cas technology opens new avenues in genetic engineering applications by increasing expediency and efficiency in the generation of desired mutations, potentially without the necessity of plasmid integration, extensive screening, or counterselection (38). So far, CRISPR-Cas9 systems have been applied in a few bacterial species to edit their genomes, with more systems rapidly becoming available. Initially, the natural dual-RNA-guided CRISPR-Cas9 was used for genome editing (19, 23), but recently, the engineered sgRNA CRISPR-Cas9 has been employed. In different approaches, the two components, Cas9 and sgRNA, can be either in two different plasmids (39) or in a single plasmid (40), and efficient genome editing has been achieved, such as deletions, insertions, and introduction of point mutations in Escherichia coli (39) and in Bacillus subtilis (40). In *Streptomyces*, a single-plasmid system with a temperature-sensitive pSG5 replicon was used for curing the plasmid after a successful series of deletions (41). Similarly, in Staphylococcus aureus, a temperature-sensitive two-vector system enables conditional recombineering and CRISPR-Cas9-mediated counterselection (42). To date, a number of studies have established CRISPR-Cas9 tools for genetic engineering in different bacteria, and this catalogue likely will continue to expand, enabling an increasing number of applications with advantages and limitations. In S. pneumoniae, a dual-RNA:Cas9 system, integrated in the chromosome, has been shown to perform insertions, deletions, or scarless single-nucleotide substitutions (19).

In this study, we have developed a replicative plasmid with a broad-host-range temperature-sensitive origin of replication carrying a concise CRISPR-Cas9-based system for advanced and markerless genome engineering in the bacterium S. pneumoniae. In particular, we demonstrate that we have successfully deleted genes and large chromosomal regions in a precise and sequential way.

The plasmid designed here has the temperature-sensitive origin of replication p*G^+^host*, which is a derivative of pWV01 of L. lactis and can be successfully propagated in pneumococcus at 30°C, while it is not stable at 37°C or 40°C. Indeed, we show that our p*G^+^host* derivative, pDS05, is rapidly lost at 37°C and at 40°C (Fig. 1; see also Fig. S1 in the supplemental material). We used this feature to eliminate the plasmid from the strains upon the desired deletion. The fact that the copies of the plasmid vary per cell does not affect our system, since even one copy of *cas9* seems to be sufficient to perform the DSB (43).

Specifically, our approach is to harness this CRISPR-Cas9 system and the homologous recombination system to perform CRISPR-Cas9-mediated counterselection. Successful transformants survive the CRISPR-Cas9-induced DSB only if they uptake the rescue HR template. At this point, we do not know whether a CRISPR-Cas9-induced DSB also promotes RecA-dependent HR with the user-defined template. Regardless, our CRISPR-Cas9 system manages to select for transformants in which single genes or even large chromosomal regions were deleted with very high efficiency. Comparing this to just performing natural transformation without counterselection, which would be an alternative for clean deletions, we show the advantages of our system (Fig. 4c). Without counterselection, we would need to screen many colonies to find correct transformants, depending on the target. This will have to be performed by colony PCR, since in most cases, the desired deletion will not give any phenotypic difference in the colonies of the successful transformant, which is a costly and time-demanding process. On the other hand, with CRISPR-Cas9-mediated counterselection, nearly all the colonies that we obtained were the desired transformant, since very few false positives have been observed.

Since we are ultimately interested to remove multiple genes and chromosomal regions from the genome, we also needed to demonstrate that our system is capable of consecutive deletions. The key for this was to easily eliminate the plasmid from the newly constructed strain. By growing the strain still carrying the plasmid at the high nonpermissive temperature, we managed to easily cure it. Next, we transformed a new plasmid and proceeded further with our deletions. Specifically, after we deleted the capsule, we next deleted virulence factors *ply* and *lytA*, proving that our CRISPR-Cas9 system has flexibility in genetic manipulation of the bacterial genome. Apart from the above-demonstrated consecutive deletions, the plasmid can be used in future studies for other genome editing options, such as SNPs, insertions, or replacements. Additionally, by including more than one sgRNA in the backbone, a higher level of multiplexing, such as concurrent or sequential deletions, can be performed.

Together, the plasmid and approach described here will be useful for the pneumococcal research community and may be applicable to other Gram-positive bacteria as well. It is likely that the zinc-inducible promoter used to drive Cas9 expression here is specific to pneumococci and would need to be exchanged by an alternative inducible promoter for the species of interest. Plasmid pDS05 is available from Addgene (157913).

## MATERIALS AND METHODS

### Bacterial strains, transformations, and growth conditions.

All pneumococcal strains used in this study are derivatives of the serotype 2 S. pneumoniae strain D39V (5, 29). All plasmids where cloned in NEB Turbo Competent E. coli (catalog number C2984; New England BioLabs). All the strains are shown in Table 1.

**TABLE 1:** Strain and plasmid list

Strain or plasmid	Relevant genotype or description	Reference(s) or source
S. pneumoniae strains		
D39V	Serotype 2 strain, wild type	5, 29
VL321	*SPV_2146* P_32_ *lacZ chl aliA*	This study
VL588	*ssbB luc kan cps*::*chl*	Lab collection
VL2172	pPEPZ read1 P_3_ BsaI *gfp* BsaI read2 N701 p7	Lab collection
VL3655	D39V(pDS05)	This study
VL3656	*SPV_2146 lacZ chl aliA*(pDS07)	This study
VL3657	Δ*lacZ*(pDS07)	This study
VL3658	Δ*lacZ*	This study
VL3659	Δ*cps*(pDS07)	This study
VL3660	Δ*cps*	This study
VL3661	Δ*cps*(pDS12)	This study
VL3662	Δ*cps* Δ*ply*(pDS12)	This study
VL3663	Δ*cps* Δ*ply*	This study
VL3664	Δ*cps* Δ*ply*(pDS13)	This study
VL3665	Δ*cps* Δ*ply* Δ*lytA*(pDS13)	This study
Plasmids		
pDS05	p*G^+^host ori*(Ts) *ermR cloDF13ori specR P_Zn_ wtcas9 P_3_ gfp* sgRNA	This study
pDS06	p*G^+^host ori*(Ts) *ermR cloDF13ori specR P_Zn_ wtcas9 P_3_* sgRNA *luc*	
pDS07	p*G^+^host ori*(Ts) *ermR cloDF13ori specR P_Zn_ wtcas9 P_3_* sgRNA *lacZ*	This study
pDS12	p*G^+^host ori*(Ts) *ermR cloDF13ori specR P_Zn_ wtcas9 P_3_* sgRNA *ply*	This study
pDS13	p*G^+^host ori*(Ts) *ermR cloDF13ori specR P_Zn_ wtcas9 P_3_* sgRNA *lytA*	This study
pRAS2	pJWV01 *P_3_*_2_ *lacZ*	Lab collection

S. pneumoniae was grown at 28°C, 30°C, 37°C, or 40°C (where indicated) without shaking in liquid C+Y medium adapted from reference 44 and containing the following compounds: adenosine (68.2 μM), uridine (74.6 μM), l-asparagine (302 μM), l-cysteine (84.6 μM), l-glutamine (137 μM), l-tryptophan (26.8 μM), casein hydrolysate (4.56 g·liter^−1^), bovine serum albumin (BSA) (729 mg·liter^−1^), biotin (2.24 μM), nicotinic acid (4.44 μM), pyridoxine (3.10 μM), calcium pantothenate (4.59 μM), thiamine (1.73 μM), riboflavin (0.678 μM), choline (43.7 μM), CaCl_2_ (103 μM), K_2_HPO_4_ (44.5 mM), MgCl_2_ (2.24 mM), FeSO_4_ (1.64 μM), CuSO_4_ (1.82 μM), ZnSO_4_ (1.58 μM), MnCl_2_ (1.29 μM), glucose (10.1 mM), sodium pyruvate (2.48 mM), saccharose (861 μM), sodium acetate (22.2 mM), and yeast extract (2.28 g·liter^−1^).

E. coli strains were cultivated in LB at 37°C with shaking. When appropriate, 100 μg/ml spectinomycin (spec) was added.

### Transformation.

To transform the different plasmid variants into S. pneumoniae, cells were grown in C+Y medium (pH 6.8) at 37°C to an optical density at 595 nm (OD_595_) of 0.1. Then, cells were treated for 12 min at 37°C with synthetic CSP-1 (100 ng ml^−1^) and incubated for 20 min at 30°C with the plasmid. After incubation, cells were grown in C+Y medium at the permissive temperature of 30°C for 120 min. S. pneumoniae transformants were selected by plating inside Columbia agar supplemented with 3% of defibrinated sheep blood (Thermo Scientific) with antibiotic selection, using erythromycin at a concentration of 0.25 μg/ml. Plates were incubated at 30°C.

To transform the HR template, cells were grown in C+Y medium (pH 6.8) at 30°C with 0.1 μg/ml erythromycin to an OD_595_ of 0.1. Then, cells were treated for 12 min at 37°C with synthetic CSP-1 (100 ng·ml^−1^) and incubated for 20 min at 30°C with the HR template. After incubation, cells were grown in C+Y medium at 30°C for 20 min. Transformants were selected by plating inside molten Columbia agar supplemented with 3% of defibrinated sheep blood (Thermo Scientific), with CRISPR-Cas9-mediated counterselection, using a mixture of 1 mM ZnCl_2_-MnSO_4_. MnSO_4_ was added to counteract potential toxicity from ZnCl_2_.

Plates were incubated at 30°C. Correct transformation was verified by PCR and sequencing. Working stocks of cells were prepared by growing cells in C+Y (pH 6.8) to an OD_595_ of 0.4. Cells were collected by centrifugation (1,595 × *g* for 10 min), resuspended in fresh C+Y medium with 15% glycerol, and stored at −80°C.

### Plasmid curing.

After the plasmid was transformed into pneumococcus and successful deletion was performed with the HR template and CRISPR-Cas9-mediated counterselection, the plasmid could be eliminated from the pneumococcal cells. To achieve that, we first grew the strain with the plasmid at the nonpermissive temperature, 40°C in C+Y, with a starting inoculum of 1/10,000. Next, we plated the liquid culture in Columbia blood agar in several dilutions, to obtain single colonies after overnight incubation at 40°C. Single colonies were screened, and 99% of them had successfully cured the plasmid from the strain.

### Recombinant DNA techniques.

Oligonucleotides were ordered from Sigma and are listed in Table 2. Phanta Max super-fidelity DNA polymerase (Vazyme) was used in PCR amplifications, restriction enzymes (Thermo Fisher Scientific) were used for digestions, and T4 DNA ligase (Vazyme) was used for ligations.

**TABLE 2:** Oligonucleotides used in this study

Name	Sequence (5′→3′)^*a*^
OVL4739_pGh F	CTCTCACACCTGCCTGTCAATCGCAACATCAAACCAAAATAAAAAC
OVL4740_pGh R	CTCTCACACCTGCCTGTTTCAAAAGCGACTCATAGAATTATTTC
OVL4741_pCDF-1b F	CTCTCACACCTGCCGTATGAATCTAGAGCGGTTCAGTAGAAAAG
OVL4742_pCDF-1b R	CTCTCACACCTGCCGTATACTTGAACGAATTGTTAGACATTATTTG
OVL4743_wtcas9 F	AGATGGCACCTGCCAGAAGTACAAGCACTTTGGGACGTTCTCCCTTAG
OVL4744_wtcas9 R	AGATGGCACCTGCCAGAACGCTAAATACGCTTCACAGTTTCTTCTTC
OVL4745_gRNA F	CTCTCACACCTGCTCACGCGTATAAGAGACAGCCATTCTACAG
OVL4746_gRNA R	CTCTCACACCTGCTCACATTGAGACAGAAAAAAAGCACCGACTC
OVL2130_GG-luc-R	AAACGGCGCCATTCTATCCTCTA
OVL2131_GG-luc-F	TATATAGAGGATAGAATGGCGCC
OVL2132_GG-lacZ-F	TATAGGATGAAGACCAGCCCTTCC
OVL2133_GG-lacZ-R	AAACGGAAGGGCTGGTCTTCATCC
OVL2142_lin pGh R	CCTAGGTCTCATATAGTTATTATACCAGGG
OVL2143_lin pGh F	GTAAGGTCTCGGTTTAAGAGCTATG
OVL2250_GG-ply-F	TATACCGAGTTGTAACAGGCAAGG
OVL2251_GG-ply-R	AAACCCTTGCCTGTTACAACTCGG
OVL2813_GG-sgRNAlytA-F	TATAAACCAAAGAAGAGTTCATGA
OVL2814_GG-sgRNAlytA-R	AAACTCATGAACTCTTCTTTGGTT
rfbD-F	TCATGACCTACCTAGCTGAAAATCG
rfbD-R+BglII	GGCCAGATCTAAGCGCCCAATAACGAAGTATATTG
P32-lacZ-BglII	ATGCAGATCTAGGCCGGCCGATATGATAAG
lacZ_R-AscI	ATCACGGGCGCGCCTTATTTTTGACACCAGACCAACTG
cam-F+AscI	ACGTGGCGCGCCAGGAGGCATATCAAATGAAC
del_CSP.dn-R	GATAGAGACGAGCTGCTGTAAGGC

a Restriction sites are underlined.

### Plasmid and strain construction.

**(i) Plasmid pDS05 [p*G^+^host ori*(Ts) *ermR cloDF13ori specR P_Zn__wtcas9 P_3_ gfp sgRNA*].** Gram-positive, temperature-sensitive origin of replication p*G^+^host* (45) and gene *ermR*, which confers resistance to erythromycin, were amplified from plasmid pGh9::ISS1 (46) using the primers OVL4739_pGh F and OVL4740_pGh R (fragment 1). Gram-negative origin of replication *cloDF13* (CDF) and gene *specR*, which confers resistance to spectinomycin, were amplified from plasmid pCDF-1b (47) with primers OVL4741_pCDF-1b F and OVL4742_pCDF-1b R (fragment 2). The gene which encodes wtCas9 under the control of the zinc-inducible promoter was amplified from plasmid pJWV102-spCas9wt (43), using the primers OVL4743_wtcas9 F and OVL4744_wtcas9 R (fragment 3). The sgRNA sequence in which the 20-base-pairing region of the sgRNA is replaced by the *gfp* gene was amplified from strain VL2172 with primers OVL4745_gRNA F and OVL4746_gRNA R (fragment 4). The four fragments were digested all together with restriction enzyme AarI and ligated. The ligation product was transformed into E. coli NEB Turbo, and transformants were selected on LB agar with spectinomycin. Correct assembly was confirmed by PCR and sequencing.

**(ii) Plasmid pDS06 [p*G^+^host ori*(Ts) *ermR cloDF13ori specR P_Zn__wtcas9 P_3_* sgRNA *luc*].** pDS05 was amplified with primers OVL2143_lin pGh F and OVL2142_lin pGh R (fragment 1). Spacer sequence of sgRNA *lacZ* was constructed by annealing primers OVL2131_GG-luc-F and OVL2130_GG-luc-R. Amplified pDS05 was digested with restriction enzyme BsaI and ligated with the annealed oligonucleotides. The ligation product was transformed into E. coli NEB Turbo, and transformants were selected on LB agar with spectinomycin. Correct assembly was confirmed by PCR and sequencing. Construction of plasmids pDS07, pDS12, and pDS13 is described in the supplemental material.

**(iii) S. pneumoniae strain VL321 *SPV_2146 P_32_ lacZ chl aliA*.** *rfbD* and *SPV_2146* were amplified from chromosomal DNA with primers rfbD-F and rfbD-R+BglII (fragment 1). *P_32_ lacZ* was amplified from pRAS2 (lab collection) with primers P32-*lacZ*-BglII and *lacZ*_R-AscI (fragment 2). Chloramphenicol resistance marker and *aliA* were amplified from strain VL588 with primers cam-F+AscI and del_CSP.dn-R (fragment 3). Fragment 1 was digested with restriction enzyme BglII, fragment 2 was digested with restriction enzymes BglII and AscI, and fragment 3 was digested with restriction enzyme AscI. All three fragments were ligated together, and the ligation product was transformed into D39V. Correct assembly was confirmed by PCR and sequencing. Construction of plasmid-containing strains VL3655, VL3656, VL3657, VL3659, VL3660, VL3661, VL3662, VL3664, and VL3665 and plasmid-cured strains VL3658, VL3660, and VL3663 is described in the supplemental material.

### Microscopy.

S. pneumoniae cells were stored as exponential-phase frozen cultures. Frozen stock was inoculated 1:100 in C+Y medium and pregrown to an OD_600_ of ∼0.1. Cells were diluted once again 1:100 in fresh C+Y (with antibiotic, if applicable) and grown to exponential phase to achieve balanced growth.

Cells were grown as described above to achieve balanced growth and subsequently concentrated and brought onto a multitest slide carrying a thin layer of 1.2% agarose in C+Y. Imaging was performed by fluorescence microscopy on a Leica DMi8 through a 100× phase-contrast lens objective (numerical aperture [NA] 1.40) with a SOLA Light Engine (Lumencor) light source. Light was filtered through external excitation filters, 470/40 nm (Chroma ET470/40x), for visualization of GFP. Light passed through a cube (Leica 11536022) with a GFP/RFP polychroic mirror (498/564 nm). External emission filters used were from Chroma ET520/40m. Images were captured using LasX software (Leica) and exported to ImageJ (48) for final preparation.

Cell outlines were detected using MicrobeJ (30). For all microscopy experiments, random image frames were used for analysis. The cell outline, object detection, and fluorescence intensity data were further processed using the R package BactMAP (31).

### Transformation efficiency assays.

To calculate the transformation efficiency, 1 μg/ml of PCR product of the DNA fragment containing the upstream and the downstream regions of the deletion target was added. Serial dilutions were plated either with or without erythromycin and with or without 1 mM ZnCl_2_-MnSO_4_, depending on the counterselection method, and the transformation efficiency was calculated by dividing the number of transformants by the total viable count. All transformation efficiency values are averages from three biologically independent experiments.

### Plating assays.

To determine the number of colonies that are resistant to erythromycin and, consequently, harbor the plasmid, cells were grown in C+Y culture, as described above. At each time point, the same amount of a dilution was plated with or without erythromycin inside Columbia blood agar, and the plates were incubated overnight at the same temperature as the liquid culture they were growing in.

### Whole-genome sequencing and variant analysis.

Genomic DNA was isolated using the FastPure Bacteria DNA isolation minikit (Vazyme) according to the manufacturer’s protocol and sent for whole-genome sequencing. Illumina library prep and sequencing were carried out by Novogene (sequencing in PE150 mode). Reads were trimmed using Trimmomatic (49) and then assembled using SPAdes (50), and contigs were reordered in Mauve (51) using D39V as a reference (29). Reads were mapped onto the scaffold using bwa (52), and read depth was determined in SAMtools (53) and plotted in R (54). To detect small variants, raw reads were mapped onto the reference using bwa, and variants were detected using Freebayes (55). Potential variants with PHRED scores greater than 30 were filtered out at a read depth per sample (DP) of >5 using vcflib (56) and then intersected with the D39V annotation using Bedtools (57).

## Supplementary Material

Supplemental file 1
