# Supplementary material for: Harnessing CRISPR-Cas9 for Genome Editing in Streptococcus pneumoniae D39V
Source: Appl Environ Microbiol. 2021 Feb 26;87(6):e02762-20. doi: 10.1128/AEM.02762-20 (PMC8105017; doi:10.1128/AEM.02762-20)
Supplement: Supplemental file 1 [file AEM.02762-20-s0001.pdf]

Supplementary Information

Harnessing CRISPR-Cas9 for genome editing in *Streptococcus pneumoniae* D39V

Dimitra Synefiaridou and Jan-Willem Veening

Supplementary Figures

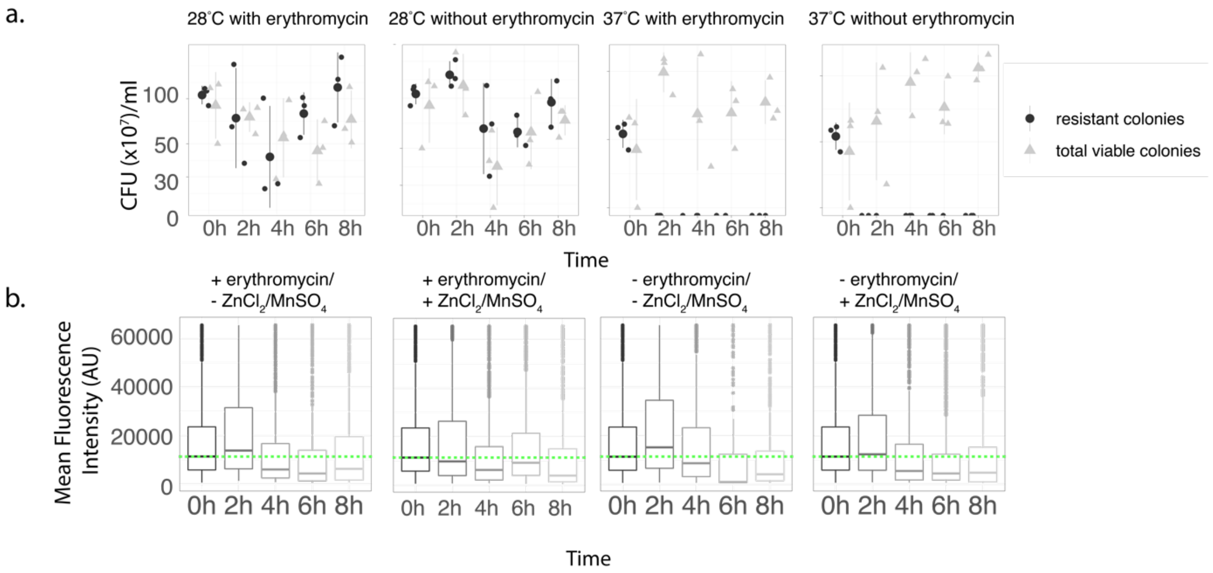

Figure S1: a. Colony forming units (CFU) count of VL3655 cells harboring plasmid pDS05 grown under four different conditions; 28°C with erythromycin, 28°C without erythromycin, 37°C with erythromycin, and 37°C without erythromycin in time points 0, 2, 4, 6 and 8 hours after dilution from the 28°C with erythromycin condition. Each time point, for each condition, a dilution of the liquid culture was plated with or without erythromycin and incubated O/N at 37°C to determine the number of resistant colonies compared to the total viable colonies. b. Quantification of mean fluorescence intensity of GFP of VL3655 cells grown under four different conditions; at 28°C: with erythromycin and without Zn^{2+}, with erythromycin and with Zn^{2+}, without erythromycin and with Zn^{2+}, and without erythromycin and with Zn^{2} in time points 0, 2, 4, 6 and 8 hours after dilution from the 28°C with erythromycin without Zn^{2+}condition. Fluorescence microscopy of ±1000 cells per condition per time point were quantified and analyzed using MicrobeJ and BactMap and plotted as box plots (box size and line represent the average intensity per cell) (see Materials and Methods). The green dotted horizontal line indicates the mean fluorescence of cells from the preculture harboring pDS05

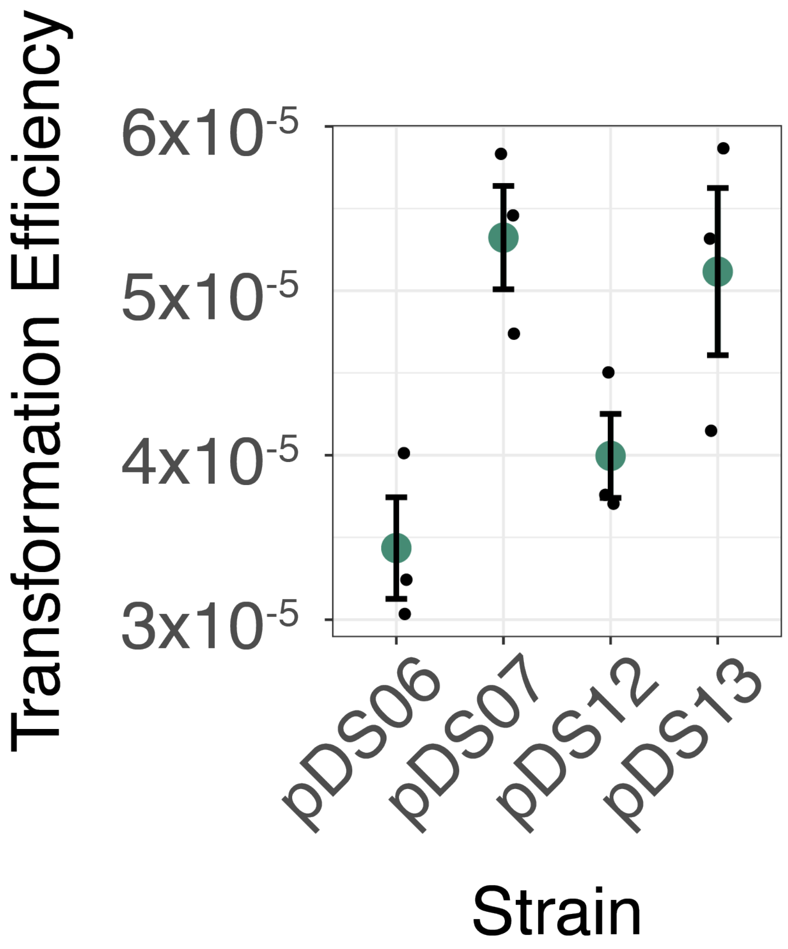

Figure S2: Transformation efficiency of four plasmids with a different sgRNA, transformed in D39V selected on erythromycin without Zn^{2+} so Cas9 is not induced. Plasmids pDS06 and pDS07 contain sgRNAs that do not target the chromosome, while pDS12 and pDS13 contain sgRNA that target the chromosome. The transformation efficiency was calculated by dividing the number of transformants by the total viable count. Black dots represent the mean of each biological replicate and the big green dot is representing the mean of the three replicates. Error bars represent the standard error of the mean

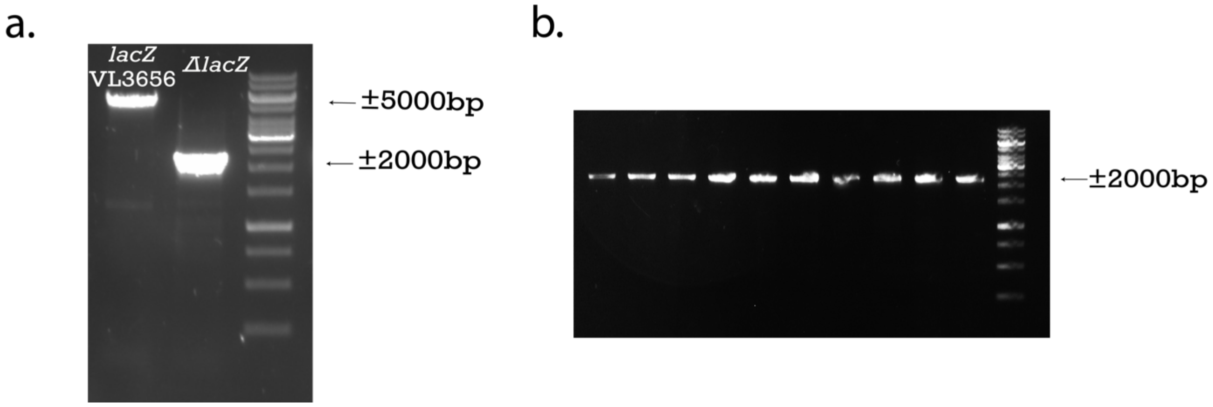

Figure S3: a. Colony PCR analysis of expected sizes. b. Eight randomly selected transformants of *lacZ* deletion.

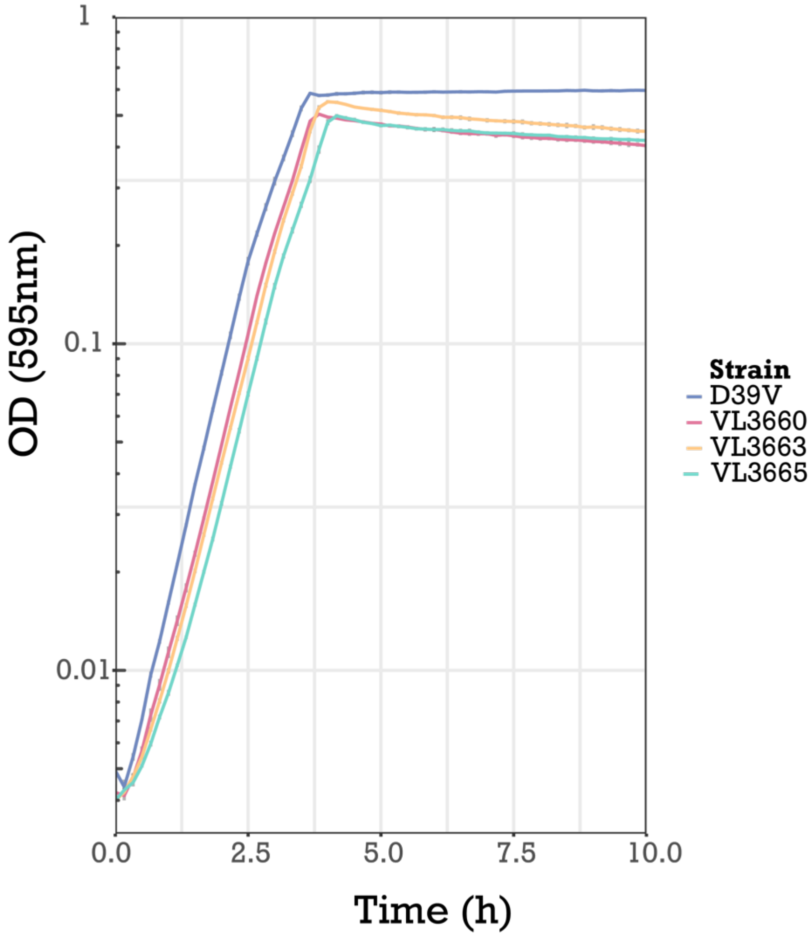

Figure S4: Cell density (OD595) of the bacterial cultures were measured every 10 min. A typical experimental outcome is shown (repeated at least 3 times). The values represent averages of three replicates. D39V = wild type, VL3660 = *Δcps*, VL3663 = *Δcps, Δply,* VL3665 = *Δcps, Δply, ΔlytA*

Supplementary methods

Plasmid and Strain construction

pDS07 (*pG⁺host ori(Ts)-ermR-cloDF13ori-specR-*P_{Zn}*_wtcas9-*P_3*_sgRNA lacZ*). pDS05 was amplified with primers OVL2143_lin pGh F and OVL2142_lin pGh R (fragment 1). Spacer sequence of *sgRNA lacZ* was constructed by annealing primers OVL2132_GG-lacZ-F and OVL2133_GG-lacZ-R. Amplified pDS05 was digested with restriction enzyme *BsaI* and ligated with the annealed oligos. The ligation product was transformed into *E. coli* NEB Turbo and transformants were selected on LB agar with spectinomycin. Correct assembly was confirmed by PCR and sequencing.

pDS12 (*pG⁺host ori(Ts)-ermR-cloDF13ori-specR-* P_{Zn} *_wtcas9-*P_3*_sgRNA ply*). pDS05 was amplified with primers OVL2143_lin pGh F and OVL2142_lin pGh R. Spacer sequence of *sgRNA ply* was constructed by annealing primers OVL2250_GG-ply-F and OVL2251_GG-ply-R. Amplified pDS05 was digested with restriction enzyme *BsaI* and ligated with the annealed oligos. The ligation product was transformed into *E. coli* NEB Turbo and transformants were selected on LB agar with spectinomycin. Correct assembly was confirmed by PCR and sequencing.

pDS13 (*pG⁺host ori(Ts)-ermR-cloDF13ori-specR-* P_{Zn} *_wtcas9-*P_3*_sgRNA lytA*). pDS05 was amplified with primers OVL2143_lin pGh F and OVL2142_lin pGh R. Spacer sequence of *sgRNA lytA* was constructed by annealing primers OVL2813_GG-sgRNAlytA-F and OVL2814_GG-sgRNAlytA-R. Amplified pDS05 was digested with restriction enzyme *BsaI* and ligated with the annealed oligos. The ligation product was transformed into *E. coli* NEB Turbo and transformants were selected on LB agar with spectinomycin. Correct assembly was confirmed by PCR and sequencing.

VL3655 (D39V + pDS05 [*pG⁺host ori(Ts)-ermR-cloDF13ori-specR-*P_{Zn}*_wtcas9-*P_3*_gfp-sgRNA*]). Plasmid pDS05 was transformed into D39V and transformants were selected on Columbia blood agar with erythromycin to produce the strain VL3655. The presence of the plasmid was confirmed by PCR and plasmid extraction.

VL3656 (*SPV_2146-lacZ-chl-aliA* + pDS07 [*pG⁺host ori(Ts)-ermR-cloDF13ori-specR-*p_{Zn}*_wtcas9-*P_3*_sgRNA lacZ*]). Plasmid pDS07, was transformed into VL321(*SPV_2146-lacZ-chl-aliA*) and transformants were selected on Columbia blood agar with erythromycin to produce the strain VL3656. The presence of the plasmid was confirmed by PCR and plasmid extraction.

VL3657 (Δ*lacZ* + pDS07 [*pG*$^{+}$*host ori(Ts)-ermR-cloDF13ori-specR-*P_{Zn}*_wtcas9-*P_{3}*_sgRNA lacZ*]). HR template Δ*lacZ* was transformed into VL3656 and transformants were selected on Columbia blood agar with $ZnCl_2$/$MnSO_4$ to produce the strain VL3657. Correct integration was confirmed by PCR.

VL3658 (*ΔlacZ*). Strain VL3657 was cured from the plasmid, as described, resulting in strain VL3658.

VL3659 (Δ*cps* + pDS07 [*pG*$^{+}$*host ori(Ts)-ermR-cloDF13ori-specR-*P_{Zn}*_wtcas9-*P_{3}*_sgRNA lacZ*]). HR template Δ*cps* was transformed into VL3656 and transformants were selected on Columbia blood agar with $ZnCl_2$/$MnSO_4$ to produce the strain VL3659. Correct integration was confirmed by PCR.

VL3660 (*Δcps*). Strain VL3659 was cured from the plasmid, as described, resulting in strain VL3660.

VL3661 (*Δcps* + pDS12 [*pG*$^{+}$*host ori(Ts)-ermR-cloDF13ori-specR-*P_{Zn}*_wtcas9-* P_{3}*-sgRNA ply*]). Plasmid pDS12 was transformed into VL3660 and transformants were selected on Columbia blood agar with erythromycin to produce the strain VL3661. The presence of the plasmid was confirmed by PCR and plasmid extraction.

VL3662 (*Δcps, Δply* + pDS12 [*pG*$^{+}$*host ori(Ts)-ermR-cloDF13ori-specR-*P_{Zn}*_wtcas9-*P_{3}*_sgRNA ply*]). HR template Δ*ply* was transformed into VL3661 and transformants were selected on Columbia blood agar with $ZnCl_2$/$MnSO_4$ to produce the strain VL3662. Correct integration was confirmed by PCR.

VL3663 (*Δcps, Δply*). Strain VL3662 was cured from the plasmid, as described, resulting in strain VL3663.

VL3664 (*Δcps, Δply* + pDS13 [*pG*$^{+}$*host ori(Ts)-ermR-cloDF13ori-specR-*P_{Zn}*_wtcas9-*P_{3}*_sgRNA lytA*]). Plasmid pDS13 was transformed into VL3663 and transformants were selected on Columbia blood agar with erythromycin to produce the strain VL3664. The presence of the plasmid was confirmed by PCR and plasmid extraction.

VL3665 (*Δcps, Δply, ΔlytA* + pDS13 [*pG*$^{+}$*host ori(Ts)-ermR-cloDF13ori-specR-* P_{Zn}*_wtcas9-*P_{3}*_sgRNA lytA*]). HR template Δ*lytA* was transformed into VL3664 and transformants were selected on Columbia blood agar with $ZnCl_2$/$MnSO_4$ to produce the strain VL3665. Correct integration was confirmed by PCR.
